# Membrane-Mediated Lateral Interactions Regulate the Lifetime of Gramicidin Channels

**DOI:** 10.3390/membranes10120368

**Published:** 2020-11-25

**Authors:** Oleg V. Kondrashov, Timur R. Galimzyanov, Rodion J. Molotkovsky, Oleg V. Batishchev, Sergey A. Akimov

**Affiliations:** Laboratory of Bioelectrochemistry, A.N. Frumkin Institute of Physical Chemistry and Electrochemistry, Russian Academy of Sciences, 31/4 Leninskiy Prospekt, 119071 Moscow, Russia; gal_timur@yahoo.com (T.R.G.); swinka87@gmail.com (R.J.M.); olegbati@gmail.com (O.V.B.)

**Keywords:** lipid membrane, gramicidin, lateral dimer, elastic deformations, theory of elasticity, channel lifetime, lateral interactions, protein-lipid interactions

## Abstract

The lipid matrix of cellular membranes is an elastic liquid crystalline medium. Its deformations regulate the functionality and interactions of membrane proteins,f membrane-bound peptides, lipid and protein-lipid domains. Gramicidin A (gA) is a peptide, which incorporates into membrane leaflets as a monomer and may form a transmembrane dimer. In both configurations, gA deforms the membrane. The transmembrane dimer of gA is a cation-selective ion channel. Its electrical response strongly depends on the elastic properties of the membrane. The gA monomer and dimer deform the membrane differently; therefore, the elastic energy contributes to the activation barriers of the dimerization and dissociation of the conducting state. It is shown experimentally that channel characteristics alter if gA molecules have been located in the vicinity of the conducting dimer. Here, based on the theory of elasticity of lipid membranes, we developed a quantitative theoretical model which allows explaining experimentally observed phenomena under conditions of high surface density of gA or its analogues, i.e., in the regime of strong lateral interactions of gA molecules, mediated by elastic deformations of the membrane. The model would be useful for the analysis and prediction of the gA electrical response in various experimental conditions. This potentially widens the possible applications of gA as a convenient molecular sensor of membrane elasticity.

## 1. Introduction

The lipid bilayer is a fundamental part of biological membranes. The amphiphilic nature of lipid molecules provides extremely low permeability of biological membranes to various polar and charged substances [1]. This allows lipid bilayers to fulfill their main barrier function. The typical thickness of a lipid bilayer is about 5 nm [2], and, in most cases, membranes can be considered as infinitely thin elastic films. Such a reduction in the space dimensions (from three dimensions to two dimensions) available for diffusing entities may have fundamental significance for the effective functioning of membrane-embedded proteins, which represent about 30% of all the proteins of eukaryotic cells [3,4]. However, there is increasing evidence that membranes are not just passive platforms serving to restrict the spatial positions of proteins or reduce the dimensionality of their accessible space; the local state of the membrane can significantly alter the mode of the membrane protein activity [5,6,7,8]. Moreover, an elasticity of lipid bilayers is considered to be responsible for an effective lateral interaction of membrane-embedded proteins or peptides [9,10,11]. Membrane inclusions or heterogeneities may induce deformations of the adjacent region of the lipid bilayer. The characteristic length of the deformation decay is typically of the order of several nanometers [12,13,14]. Thus, for far separated inclusions, the deformations induced by them are independent, and the elastic energy is additive. Upon mutual approaching, the deformations start to overlap, leading to effective lateral interaction of the proteins or peptides, incorporated into the lipid membrane. In numerous theoretical works, it is shown that such inclusions can repulse [9], attract [15] or interact with each other in a complex non-monotonous manner [9,10,12,16], depending on the type of inclusion and on the particular elasticity model applied to evaluate the energy of the membrane-mediated interactions. Though such interactions can be predicted based on general physical principles, they are hardly observable experimentally; at least, it is not trivial to prove that it is precisely deformations of the membrane that are responsible for lateral interactions of membrane inclusions. There is, however, a special type of membrane inclusions, which is well-suited for the measurement of the local elastic stress of the membrane: mechanosensitive ion channels [17,18]. Probably the most studied mechanosensitive channel is the bacterial large conductance mechanosensitive ion channel (MscL), the gating of which is triggered by the lateral tension of the membrane [17]. MscL is a large protein with 10 transmembrane regions. Thus, its production and reconstitution into the model lipid membrane system are not trivial. Gramicidin A (gA) is another example of an ion channel whose electrical response has been proven to strongly depend on local elastic properties of the membrane [19,20,21,22]. gA consists of 15 alternating L and D amino acids, and when incorporated into a lipid membrane, it usually retains a *β*^6.3^-helical secondary structure [23,24,25,26]. The ion-conducting channel is formed by a transmembrane head-to-head dimer of two molecules of gA; the dimerization is driven by the formation of 4–6 H-bonds at monolayer interface [23,27]. Electrical activity of the channel is characterized by the probability of formation of the conducting state and by its average lifetime [21,28]. Both parameters are shown to strongly depend on elastic properties of the membrane, in particular, its thickness [20,29], spontaneous curvature [19] and lateral tension [28,30]. Due to high sensitivity and fast electrical response, gA is widely used as a molecular mechanical sensor [22].

Membrane-mediated interactions of gA (monomer-monomer, monomer-dimer, dimer-dimer) should lead to cooperative effects and the non-ideality of the gA ensemble, observed as dependence of channel lifetime and formation probability on the local surface density of gA monomers. In order to quantitatively study such interactions, one should be able to set the distance between interacting gA molecules in a controllable manner. This can be achieved by using so-called lateral dimers, where two gA monomers located in the same lipid monolayer of the membrane are connected by a linker. In the works [31,32,33], the linkers used to form lateral dimers are peptide- [32], ethylene glycol- [33], or (strept)avidin-biotin pair-based [31]. These linkers possess a different length and flexibility. However, it is obtained that in most cases lateral dimers form so-called tandem channels [31] of approximately doubled conductivity characterized by extremely long lifetimes that exceed those of regular gA channels by about 1000 times [31,32,33]. Tandem channels are not formed in case of a relatively short and rigid linker [32]; moreover, the tandem channel lifetime depends on the lipid bilayer thickness: the larger the thickness, the longer the lifetime [33].

In the present work, we utilized the theory of elasticity of lipid membranes to develop a model of gA activity and lateral interactions, explicitly considering elastic deformations of the membrane induced by gA monomers, coaxial pairs and lateral dimers. We quantitatively evaluated the contribution of elastic deformations into the activation energies of channel formation (transbilayer dimerization) and dissociation. We obtained that deformations arising in the vicinity of the lateral dimer substantially increase the energy barrier of channel dissociation, thus providing an exponential growth of the channel lifetime.

## 2. Materials and Methods

**Figure 1:**
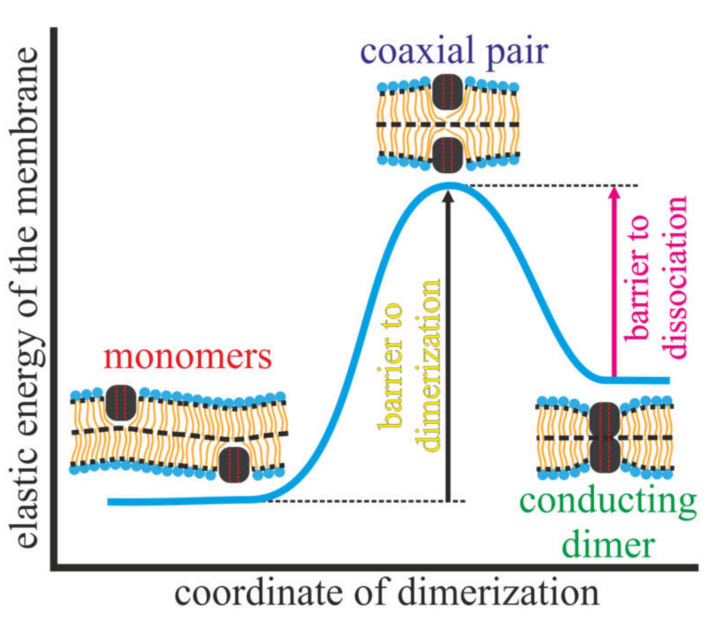
Possible configurations of two gramicidin A molecules located in opposing monolayers of the membrane: far separated monomers (left); conducting dimer (right); coaxial pair (middle). The elastic energy of the membrane in these configurations is shown schematically; the energy barrier of dimerization is the difference of the energies of the pair and two monomers; the energy barrier of dissociation is the difference of the energies of the pair and dimer. Pairs of vertical red dashed lines on gA molecules schematically show the pore inside them.

The lifetime of the conducting state of gA is determined by the energy barrier for the dissociation of the conducting dimer, while the probability of the channel formation is determined by the energy barrier to the association (dimerization). Both processes, dimerization of initially far separated monomers to conducting dimer and dissociation of the conducting dimer into two monomers, are supposed to pass through the common intermediate state of two coaxial monomers, which is referred to as a “pair” [16]. The state of the pair corresponds to the top of the energy barrier, separating the conducting state (dimer) and the state of the two isolated (far separated) monomers (Figure 1).

The energy barrier of dimerization is, thus, determined as the difference of the energies of the pair and two far separated monomers; the energy barrier of dissociation is the difference of the energies of the pair and dimer. Note that the energy barrier of dissociation is determined up to the energy of 4–6 H-bonds formed between two dimerizing gA monomers at the membrane midplane [23,27]. However, the energy of H-bonds is constant, i.e., it is independent on the presence of other gA molecules in the vicinity of the conducting dimer, on membrane elastic properties, etc.

**Figure 2:**
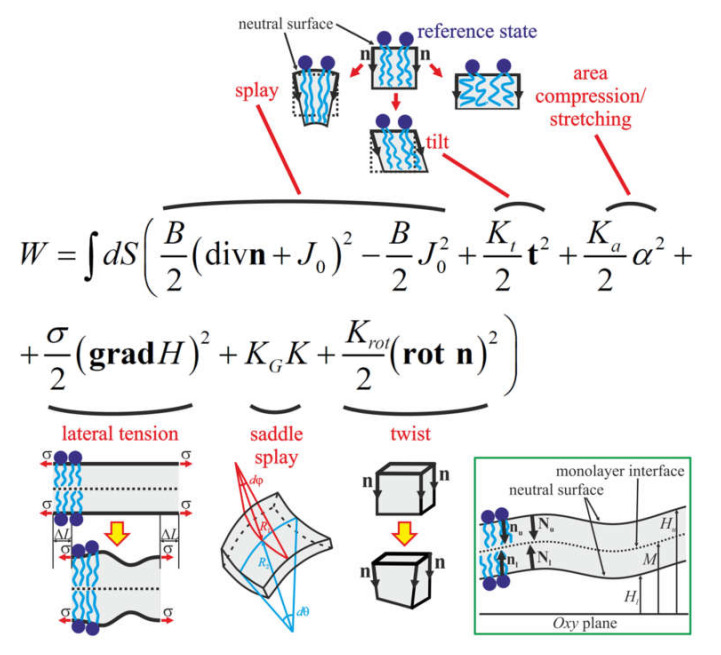
Schematic illustration of membrane deformations. In the reference state, directors, **n**, are parallel to each other and to normals of the neutral surface. Upon splay deformation, an angle arises between directors at two adjacent points of the neutral surface. Tilt deformation corresponds to a deviation of directors from normals. Upon lateral compression-stretching, the area of the neutral surface changes while the orientation of directors and normals remains unaltered. The contribution of the lateral tension arises as the projected area of the neutral surface decreases upon deformations that require pulling additional material from an inventory lipid reservoir, thus performing a work against the lateral tension, *σ*. Saddle splay accounts for Gaussian curvature of the lipid monolayer surface, which is a product of two principle curvatures of the surface, 1/(*R*_1_*R*_2_). Twist deformation is determined by **rot**(**n**). The inset in the green rectangle illustrates the directors and normals; functions *H_u_*(*x*, *y*), *M*(*x*, *y*) and *H_l_*(*x*, *y*) describe the membrane shape.

In each configuration of gramicidin A, the membrane is deformed in its vicinity. To calculate the energy and lateral distribution of deformations, we utilize the continuous theory of elasticity originally developed in the work [34] and further generalized by accounting for additional deformational modes [11,12,16]. In the framework of this theory, the average orientation of lipid molecules is described by the vector field of unit vectors called directors, **n**. The field of directors is set at some surface called dividing surface, which lies inside a lipid monolayer parallel to its external polar surface. The shape of the dividing surface is described by the vector field of its unit normals, **N**. We introduce a Cartesian coordinate system *Oxyz*, and direct its *Oz* axis perpendicularly to the undisturbed dividing surface, which is considered flat in the absence of gramicidin A embedded into the membrane. The origin *O* is placed at the intermonolayer surface of the lipid bilayer. The monolayer in the half-space *z* > 0 is called the upper, for definiteness, and the opposite monolayer is called the lower. The variables corresponding to the upper monolayer are denoted by the index “*u*”; those corresponding to the lower one by the index “*l*.” The shape of the dividing surface of the upper and lower monolayers is described by functions *H_u_*(*x*, *y*) and *H_l_*(*x*, *y*), which are the *z*-coordinates of the corresponding surface at the point (*x*, *y*). In case of small deformations, such a specification uniquely determines the surface. The *z*-coordinate of the monolayer interface is denoted by the function *M*(*x*, *y*) (Figure 2, inset). We consider the following deformations of a lipid monolayer [34,35]: (i) splay, which is characterized by the angle between the directors in adjacent points of the dividing surface; quantitatively, its contribution is given by the divergence of the director along the dividing surface, div(**n**); (ii) tilt, which is characterized by the deviation of the director from the normal vector at a given point of the dividing surface; quantitatively, its contribution is determined by the tilt-vector **t** = **n**/(**nN**)—**N** ≈ **n**—**N**; (iii) lateral stretching-compression, which is characterized by the local relative deviation of the area of the dividing surface *α* = (*a* − *a*_0_)/*a*_0_, where *a* and *a*_0_ are the current and initial area per lipid molecule at the dividing surface, respectively; (iv) lateral tension of the membrane, which accounts for the difference of the projected area of the dividing surface onto the *Oxy* plane before and after the deformation; quantitatively, it is given by the deviation of the dividing surface from the plane of its initial state, **grad**(*H*); (v) saddle splay, which in the Cartesian coordinate system can be written as $K=\frac{\partial n_{x}}{\partial x}\frac{\partial n_{y}}{\partial y}-\frac{\partial n_{x}}{\partial y}\frac{\partial n_{y}}{\partial x}$ (*n_x_* and *n_y_* are projections of the director onto *Ox* and *Oy* axes, respectively); (vi) twist, which is characterized by the **rot**(**n**). The deformations are deemed small and the energy is calculated in quadratic order on them [11,12,16]:

(1) $$\begin{array}{ll} W & =\int dS\left(\frac{B}{2}{\left(\mathrm{div}\left(n\right)+J_{0}\right)}^{2}-\frac{B}{2}J_{0}^{2}+\frac{K_{t}}{2}t^{2}+\frac{\sigma }{2}{\left(grad\left(H\right)\right)}^{2}\right.+\\ & \left.+\frac{K_{a}}{2}\alpha^{2}+K_{G}K+\frac{K_{rot}}{2}{\left(rot\left(n\right)\right)}^{2}\right), \end{array}$$

where *B* is the splay, *K_t_* is the tilt, *K_a_* is the lateral stretching-compression, *K_G_* is the saddle splay moduli of the monolayer, *K_rot_* is the twist modulus, *σ* is the lateral tension of the monolayer and *J*_0_ is the spontaneous curvature of the lipid monolayer. The integration in Equation (1) is performed over the dividing surface of the monolayer. We relate the deformations and the elastic moduli to a specific dividing surface, referred to as the neutral surface, where the deformations of splay and lateral stretching-compression are energetically decoupled. The neutral surface is experimentally shown to exist; it lies in the region of the junction of polar heads and hydrophobic tails of a lipid monolayer, about 0.7 nm deep from its external polar surface [36]. The deformations are schematically illustrated in Figure 2. Note that in many works on membrane elasticity, bending and splay deformations are often used as synonyms, although these deformations are defined differently: splay deformation reflects the change of lipid orientation with respect to each other, characterized by a divergence of the director field div(**n**) [34], while bending is just a curving of the membrane leaflet surface, characterized by its mean curvature, which is equal to divergence of the vector field of unit normals to the surface of the lipid monolayer, div(**N**). However, splay and bending deformations are equivalent at zero tilt deformation, **t** = **n − N** = **0**. Indeed, in this case, the membrane normal equals the director, and thus, div(**n**) = div(**N**), and the bending (curving) of the membrane monolayer surface leads to the equivalent splay of lipid molecules. It has been shown [34] that splay and tilt deformations are independent, and the “traditional” bending modulus equals the splay rigidity.

The bulk modulus of lipid membranes is extremely high, about 10^10^ J/m^3^ [37]. This allows considering the hydrophobic part of a lipid monolayer as a locally incompressible medium, which means that the volume of the monolayer element does not change upon deformations. Within the required accuracy, the condition of local volumetric incompressibility reads [11,12,16]:

(2) $$H_{u}-M=h-\frac{h^{2}}{2}\mathrm{div}\left(n_{u}\right)-h\alpha_{u},\;M-H_{l}=h-\frac{h^{2}}{2}\mathrm{div}\left(n_{l}\right)-h\alpha_{l},$$

for the upper and lower monolayers, respectively. In Equation (2) *h* is the thickness of the hydrophobic part of a lipid monolayer in its planar undisturbed state; for simplicity, we refer to *h* as the monolayer thickness. For small deformations up to the first order of smallness, $n_{u,l}=\left(n_{u,l}{}_{x}\left(x,y\right),n_{u,l}{}_{y}\left(x,y\right),\mp 1\right),$$N_{u,l}=\left(\pm \partial H_{u,l}\left(x,y\right)/\partial x,\pm \partial H_{u,l}\left(x,y\right)/\partial y,\mp 1\right),$ where the upper signs are for the director and normal projections of the upper monolayer and the lower signs are for the lower monolayer, respectively. Thus, within the required accuracy, we have for the vectors **N***_u_*, **N***_l_* the expressions: $N_{u,l}=\pm grad\left(H_{u,l}\right).$ Furthermore, we take into account that for small deformations **t** ≈ **n**–**N**, and thus, $t_{u,l}=n_{u,l}\mp grad\left(H_{u,l}\right)$. Using such notations, expressing *α_u_*_,*l*_ from Equation (2) and substituting it into the energy functional, Equation (1), we obtain the energy functional of a deformed lipid bilayer:

(3) $$\begin{array}{l} W=\int dS_{u}(\frac{B}{2}{\left(\mathrm{div}(n_{u})+J_{0}\right)}^{2}-\frac{B}{2}J_{0}^{2}+\frac{K_{t}}{2}{\left(n_{u}-grad(H_{u})\right)}^{2}+\\ +\frac{\sigma }{2}{\left(grad\left(H_{u}\right)\right)}^{2}+\frac{K_{a}}{2h^{2}}{\left(h-\frac{h^{2}}{2}\mathrm{div}\left(n_{u}\right)+M-H_{u}\right)}^{2}+\\ \left.+K_{G}K_{u}+\frac{K_{rot}}{2}{\left(rot\left(n_{u}\right)\right)}^{2}\right)+\\ +\int dS_{l}\left(\frac{B}{2}{\left(\mathrm{div}\left(n_{l}\right)+J_{0}\right)}^{2}-\frac{B}{2}J_{0}^{2}+\frac{K_{t}}{2}{\left(n_{l}+grad\left(H_{l}\right)\right)}^{2}\right.+\\ +\frac{\sigma }{2}{\left(grad\left(H_{l}\right)\right)}^{2}+\frac{K_{a}}{2h^{2}}{\left(h-\frac{h^{2}}{2}\mathrm{div}\left(n_{l}\right)-M+H_{l}\right)}^{2}+\\ \left.+K_{G}K_{l}+\frac{K_{rot}}{2}{\left(rot\left(n_{l}\right)\right)}^{2}\right). \end{array}$$

In order to correctly set the problem of the functional optimization, one should impose appropriate boundary conditions. We require the membrane to be unperturbed at infinity (far from the inclusions):

(4) $$n_{u,l}\left(\infty \right)={\left(0,0,\mp 1\right)}^{T},\;M\left(\infty \right)=0,\;H_{u}\left(\infty \right)=h,\;H_{l}\left(\infty \right)=-h.$$

We require a continuity of directors and neutral surfaces of the upper and lower monolayers everywhere except the inclusions. At the inclusion we set specific boundary conditions. The intersection of the gramicidin A dimer or coaxial pair with the neutral surface of either the upper or lower monolayer can be considered as a circle of the radius *r*_0_, the center of which lies at the dimer or pair vertical axis. Let us denote this circular contour as Γ. Then, the dimer sets the following condition at its boundary:

(5) $$H_{u}\left(\Gamma \right)-H_{l}\left(\Gamma \right)=2h_{p},$$

where *h_p_* is the length of the hydrophobic part of the gA monomer, measured along its vertical axis. At the boundary of the coaxial pair or monomers of gA, we impose the conditions [12,16]:

(6) $$\begin{array}{l} n_{u,n}\left(\Gamma \right)=n_{l,n}\left(\Gamma \right)=-\frac{h-h_{p}}{\sqrt{{\left(h-h_{p}\right)}^{2}+h_{p}^{2}}},\;n_{u,t}\left(\Gamma \right)=n_{l,t}\left(\Gamma \right)=0,\\ H_{u}\left(\Gamma \right)=H_{m}=const,\;H_{l}\left(\Gamma \right)=-H_{m}=const \end{array}$$

where *n_n_* and *n_t_* are the normal and tangential components of the director, respectively, as determined with respect to the circle Γ; *H_m_* is the constant, which is determined from the minimization of the energy functional Equation (3). In case when one monomer (e.g., in the upper monolayer) is tilted with respect to the vertical axis by a horizontal vector Δ**n**, the boundary conditions Equation (6) should be modified as follows:

(7) $$n_{u}\left(\Gamma \right)=n_{0}\left(\Gamma \right)+\Delta n,\;H_{u}\left(\Gamma \right)=H_{m}+\Delta r\Delta n,$$

where Δ**r** = **r** – **r**_0_; Γ is the tilted circle of the intersection of the boundary of the gA with the monolayer neutral surface, **r_0_** is the radius-vector of its center, **r** is the radius vector, which sets a point at the circle Γ, and **n**_0_ is the boundary director for the coaxial pair (Equation (6)). In case of the tilted pair, one should apply the conditions of Equation (7) to both monomers of the pair.

Minimization of the energy functional Equation (3) under the conditions of Equations (4)–(7) allows one to obtain the energy of the corresponding gramicidin A configuration (dimer, pair or monomer). In order to simultaneously take into account several dimers/pairs/monomers in the membrane, it is necessary to explicitly set the boundary conditions Equations (5)–(7) for each dimer/pair/monomer.

The energy functional Equation (3) can be minimized analytically only for rotationally symmetric configurations of gA: one dimer, one coaxial pair or one monomer. For the systems of two dimers, a dimer and pair, a dimer and monomer and a pair and monomer, the analytical minimization is impossible. In these cases, we minimized the energy functional numerically utilizing the finite element method with an adaptive mesh.

We divided the neutral surfaces of the lipid monolayers into triangles, in each of which we replaced the deformation fields with polynomials of the first degree in coordinates. In other words, we replaced the smooth deformation functions with their first-order interpolants, built on the nodes of the computational mesh. Since Equation (3) does not contain derivatives higher than the first order, such an interpolation is sufficient. Furthermore, we integrated the energy density in Equation (3) over the area of an elementary triangle with the coordinates of the vertices (*x*_1_, *y*_1_), (*x*_2_, *y*_2_) and (*x*_3_, *y*_3_):

(8) $$\begin{array}{ll} \Delta W & =\int_{\Delta S\left(x_{1},y_{1},x_{2},y_{2}xl3\right),y_{3}}\left(a_{1}x+b_{1}y+c+a_{2}x^{2}+b_{2}y^{2}+dxy\right)dS=\\ & =\frac{\left|x_{2}y_{1}-x_{3}y_{1}-x_{1}y_{2}+x_{3}y_{2}+x_{1}y_{3}-x_{2}y_{3}\right|}{24}\times \\ & \times \left(12c+2a_{2}\sum_{i,j=1}^{3}x_{i}x_{j}\right.+2b_{2}\sum_{i,j=1}^{3}y_{i}y_{j}+\\ & \left.+4a_{1}\sum_{i=1}^{3}x_{i}+4b_{1}\sum_{i=1}^{3}y_{i}+d\left(\sum_{i,j=1}^{3}x_{i}y_{j}+\sum_{i=1}^{3}x_{i}y_{i}\right)\right), \end{array}$$

where *a*_1_, *a*_2_, *b*_1_, *b*_2_, *c* and *d* are the corresponding expansion coefficients of the energy density in Equation (3) upon substitution of the interpolants. Assuming that the deformations on the triangle can be represented as *f*(*x*, *y*) = α[*f*]*x* + β[f]*y* + *γ*[*f*], where *f* is the deformation field (*n_ux_*, *n_uy_*, etc.), we obtained for the coefficients *a*_1_, *a*_2_, *b*_1_, *b*_2_, *c* and *d* expressions in the following explicit forms:

$$\begin{array}{ll} a_{1} & =K_{a}\frac{\alpha [H_{1}]-\alpha [M]}{2h^{2}}\left(2\gamma [H_{1}]-2\gamma [M]-2h+\alpha [n_{ux}]h^{2}+\beta [n_{uy}]\right)+\\ & +K_{t}\left(\alpha [n_{ux}]\gamma [n_{ux}]+\alpha [n_{uy}]\gamma [n_{uy}]-\alpha [H_{1}]\alpha [n_{ux}]-\beta [H_{1}]\alpha [n_{uy}]\right), \end{array}$$

(9) $$\begin{array}{ll} b_{1} & =K_{a}\frac{\beta [H_{1}]-\beta [M]}{2h^{2}}\left(2\gamma [H_{1}]-2\gamma [M]-2h+\alpha [n_{ux}]h^{2}+\beta [n_{uy}]\right)+\\ & +K_{t}\left(\beta [n_{ux}]\gamma [n_{ux}]+\beta [n_{uy}]\gamma [n_{uy}]-\alpha [H_{1}]\beta [n_{ux}]-\beta [H_{1}]\beta [n_{uy}]\right), \end{array}$$

$$\begin{array}{ll} c & =\frac{B}{2}{\left(\alpha [n_{ux}]+\beta [n_{uy}]\right)}^{2}+\\ & +K_{a}\frac{2\gamma [H_{1}]-2\gamma [M]-2h+h^{2}\left(\alpha [n_{ux}]+\beta [n_{uy}]\right)}{8h^{2}}+\\ & +\frac{K_{\mathrm{rot}}}{2}{\left(-\alpha [n_{uy}]+\beta [n_{ux}]\right)}^{2}+\frac{\sigma }{2}\left(\alpha {\left[H_{1}\right]}^{2}+\beta {\left[H_{1}\right]}^{2}\right)+\\ & +\frac{K_{t}}{2}\left(\alpha {\left[H_{1}\right]}^{2}+\beta {\left[H_{1}\right]}^{2}+\gamma {\left[n_{ux}\right]}^{2}+\gamma {\left[n_{uy}\right]}^{2}-\right.\\ & \left.-2\alpha [H_{1}]\gamma [n_{ux}]-2\beta [H_{1}]\gamma [n_{uy}]\right), \end{array}$$

$$\begin{array}{ll} a_{2} & =K_{a}\frac{{\left(\alpha [H_{1}]-\alpha [M]\right)}^{2}}{2h^{2}}+\frac{K_{t}}{2}\left(\alpha {\left[n_{ux}\right]}^{2}+\alpha {\left[n_{uy}\right]}^{2}\right),\\ b_{2} & =K_{a}\frac{{\left(\beta [H_{1}]-\beta [M]\right)}^{2}}{2h^{2}}+\frac{K_{t}}{2}\left(\beta {\left[n_{ux}\right]}^{2}+\beta {\left[n_{uy}\right]}^{2}\right),\\ d & =K_{a}\frac{\left(\alpha [H_{1}]-\alpha [M]\right)\left(\beta [H_{1}]-\beta [M]\right)}{h^{2}}+\\ & +K_{t}\left(\alpha [n_{ux}]\beta [n_{ux}]+\alpha [n_{uy}]\beta [n_{uy}]\right). \end{array}$$

**Figure 3:**
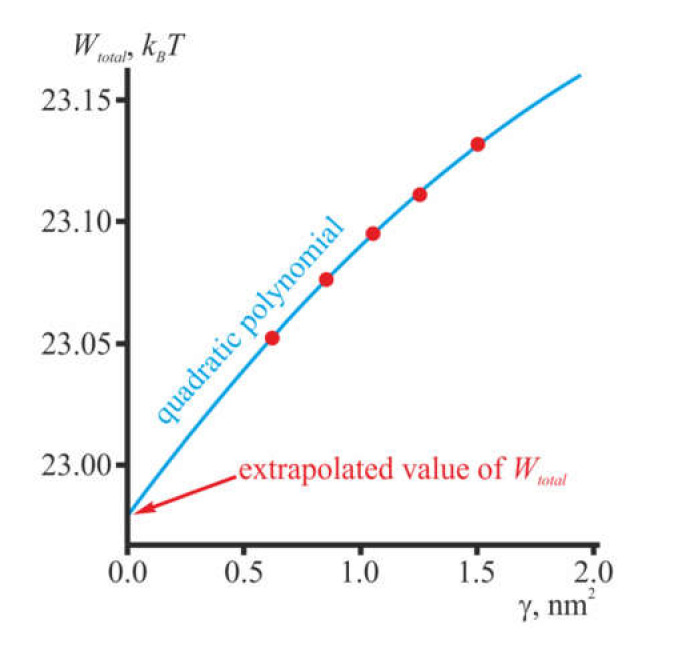
Representative plot of the approximation of *W_total_* for *γ* → 0. *W_total_* was explicitly calculated for five mesh sizes *γ_i_* (shown as red circles). The dependence *W_total_*(*γ_i_*) was approximated by a quadratic polynomial (blue curve), and then extrapolated to *γ* = 0. The extrapolated values *W_total_*(*γ* = 0) for different configurations of gramicidin A are referred to as the elastic energies of the membrane in these configurations throughout the text below. This particular plot was obtained for heterodimeric configuration of gramicidin A in the dioleoylposphatidylcholine (DOPC) membrane; the position of the single gA molecule was *x* = 0.4 nm, *y* = 0 (see the text below for details).

We expressed the expansion coefficients in terms of values at the vertices of the elementary triangles with the coordinates of the vertices (*x*_1_, *y*_1_), (*x*_2_, *y*_2_) and (*x*_3_, *y*_3_):

(10) $$\begin{array}{l} \alpha [f]=\frac{f_{2}y_{1}-f_{3}y_{1}-f_{1}y_{2}+f_{3}y_{2}+f_{1}y_{3}-f_{2}y_{3}}{x_{2}y_{1}-x_{3}y_{1}-x_{1}y_{2}+x_{3}y_{2}+x_{1}y_{3}-x_{2}y_{3}},\\ \beta [f]=-\frac{f_{2}x_{1}-f_{3}x_{1}-f_{1}x_{2}+f_{3}x_{2}+f_{1}x_{3}-f_{2}x_{3}}{x_{2}y_{1}-x_{3}y_{1}-x_{1}y_{2}+x_{3}y_{2}+x_{1}y_{3}-x_{2}y_{3}},\\ \gamma [f]=\frac{f_{3}x_{2}y_{1}-f_{2}x_{3}y_{1}-f_{3}x_{1}y_{2}+f_{1}x_{3}y_{2}+f_{2}x_{1}y_{3}-f_{1}x_{2}y_{3}}{x_{2}y_{1}-x_{3}y_{1}-x_{1}y_{2}+x_{3}y_{2}+x_{1}y_{3}-x_{2}y_{3}}, \end{array}$$

where *f_i_* = *f*(*x_i_*, *y_i_*) denotes the values of the deformation fields at the mesh nodes. We substituted such expressions into Equation (9), and took the sum over all triangular regions occupied by the lipid monolayer. As a result, we obtained the elastic energy of one (e.g., upper) monolayer as a function of the values of the deformation fields at the mesh nodes. Performing similar calculations for the opposite (i.e., lower) monolayer, we obtained the total elastic energy, *W_total_*, of the membrane as a sum of the elastic energies of its constituent monolayers. *W_total_* depends on the values of the deformation fields specified at the mesh nodes. In order to find the numerical value of the energy of the elastic deformations, we minimized the function *W_total_* over all values of the deformation fields at the mesh nodes, except for those specified by the boundary conditions in Equations (4)–(7). As an effective infinitely distant boundary (see Equation (4)), we used a rectangle, the sides of which were at least 25 nm away from the peptides; such a distance substantially exceeds the characteristic length of the deformation decay, which is about several nanometers [12,13,14]. As the deformations are non-zero only in a relatively small area in the vicinity of the peptides, we used inhomogeneous meshes, of which the surface density of nodes increased when it approached the peptide boundary. In turn, the peptide boundary was represented in a piecewise linear approximation. In order to improve the accuracy of the calculation, the neutral surfaces around the peptide(s) were divided into several regions. Each region was determined by the inequality *r_i_*_– 1_ ≤ *d* ≤ *_ri_*, where *d* is the distance to the boundary of the nearest peptide and *r_i_*_- 1_, *r_i_* are constants defining inner and outer boundaries of the regions, respectively, for *i* = 1, 2, 3, 4, 5. The particular values of *r_i_* were: *r*_0_ = 0; *r*_1_ = 1 nm; *r*_2_ = 1.5 nm; *r*_3_ = 4 nm; *r*_4_ = 11 nm; *r*_5_ = ∞. Assuming that the maximum area of an elementary triangle of the computational mesh is 0.5*γ* (in nm^2^), we divided the regions defined above into elementary triangles in such a way that the area of each triangle did not exceed *γθ_i_*, where *θ_i_* was *θ_i_* = 0.01, 0.02, 0.04, 0.05, 0.5 for the *i*th region. This procedure allowed obtaining the value of *W_total_* for each fixed value of *γ*. Obviously, the smaller the *γ*, the closer the obtained value *W_total_* should be to the true minimum of the elastic energy functional (3). To diminish the effect of the finite size of the mesh, we calculated at least five values of the elastic energy *W_total_* of the system for sequentially decreasing meshes. After that, we applied a quadratic extrapolation to the zero size (*γ* → 0) of the computational mesh, similarly to the works [12,16]. We used the following particular values of *γ*: 0.62; 0.85; 1.05; 1.25; 1.5. A quadratic polynomial was used as an approximating function. The coefficients of the polynomial were determined based on the obtained values of *W_total_*(*γ_i_*). A representative plot of the approximation is shown in Figure 3. It can be seen that the error caused by the finite size of the computational mesh elements is typically orders of magnitude less than the calculated elastic energies *W_total_*.

In the work [33], membranes of the following compositions were used: dimiristoylphosphatidylcholine (DMPC) + 20 mol% cholesterol, dipalmitoylphosphatidylcholine (DPPC), dioleoylposphatidylcholine (DOPC) and diphytanoylphosphatidylcholine (DPhPC). However, activity of tandem channels is mostly pronounced in the relatively thick bilayers of DOPC and DPhPC. DMPC bilayers are quite thin and relatively unstable; DPPC bilayers are in a gel phase at room temperature, which is not usual for “conventional” membranes. In the paper [32], membranes made from DPhPC were used, except experiments on circular dichroism, which were carried out on DMPC vesicles. In the work [31], experiments were performed on membranes made from DPhPC. Thus, to obtain quantitative results, we used elastic parameters typical for membranes made from DOPC and DPhPC: splay modulus *B* = 0.42 × 10^–19^ J ≈ 10 *k_B_T* for DOPC [38] and *B* = 14 *k_B_T* for DPhPC [39]; spontaneous curvature *J*_0_ = −0.091 nm^–1^ for DOPC [40] and *J*_0_ = −0.2 nm^–1^ for DPhPC (as far as we know, this value was not determined experimentally; we estimated it from the chemical structure of DPhPC, as its spontaneous curvature should be somewhat more negative than for DOPC); the thickness of the hydrophobic part of monolayer *h* = 1.45 nm for DOPC [38,41] and *h* = 1.4 nm for DPhPC. Other elastic parameters were either taken equal for DOPC and DPhPC membranes, or were related with the listed above parameters in a universal way: tilt modulus *K_t_* = 40 mN/m ≈ 10 *k_B_T*/nm^2^ [34]; lateral compression-stretching modulus *K_a_* = 133 mN/m ≈ 32 *k_B_T*/nm^2^ [38]; Gaussian modulus *K_G_* = –0.5*B* [42]; lateral tension *σ* = 0.1 mN/m ≈ 0.025 *k_B_T*/nm^2^; twist modulus *K_rot_* = *B*/2 [12]; *h_p_* = 0.75 nm; *r*_0_ = 1 nm [16]. All values are given per one monolayer.

## 3. Results

**Figure 4:**
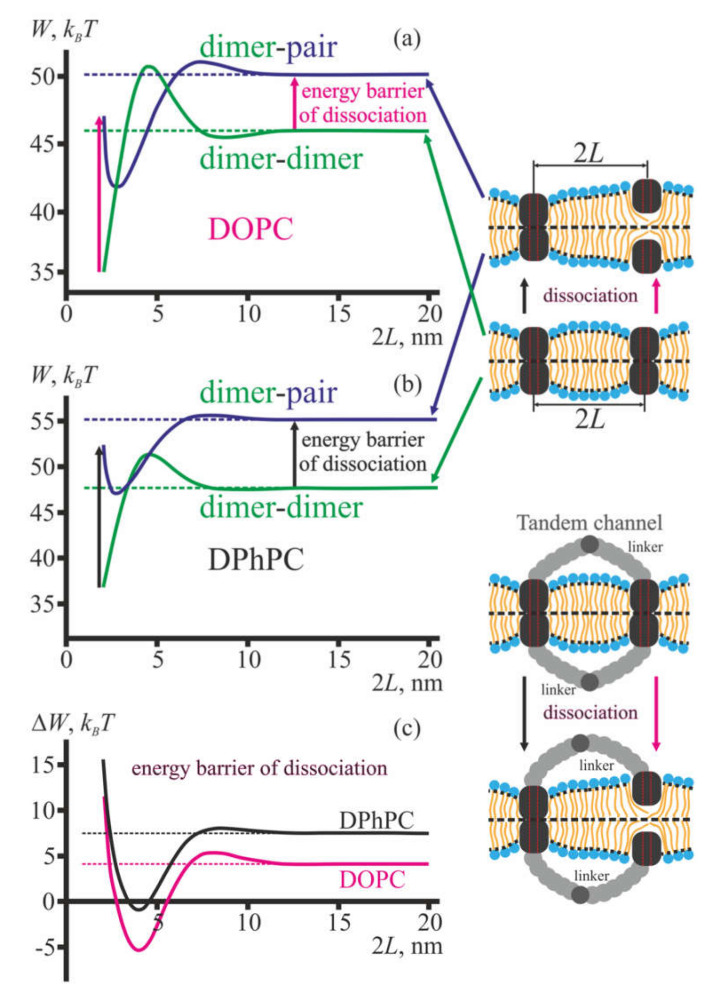
The energy of membrane elastic deformations induced by the incorporated gramicidin A. Dependence of the membrane elastic energy on the distance between two gA dimers (green curve) and gA dimer and coaxial pair (blue curve) for membranes formed from DOPC (**a**) and diphytanoylphosphatidylcholine (DPhPC) (**b**). (**c**) Dependence of the elastic contribution to the energy barrier of dissociation of gA conducting state (dimer) on the distance between two conducting dimers; the dependences are obtained as a difference of blue and green curves in the plots (**a**) and (**b**) for membranes formed from DOPC (magenta curve) and DPhPC (black curve), respectively. The distances are measured between vertical axes of revolution of the molecules. Pairs of vertical red dashed lines on gA molecules schematically show the water pore inside them. Different gramicidin A configurations are schematically shown in the right row. The nearest possible distance between two dimers or a dimer and a pair equals to 2*r*_0_ = 2 nm.

Figure 4a,b illustrate the calculated dependences of the membrane elastic energy on the distance 2*L* between the axes of two dimers (green curve) and a dimer and coaxial pair (blue curve) of gA for membranes formed from DOPC (Figure 4a) and DPhPC (Figure 4b). The tandem channel is formed by two lateral dimers of gA located in the opposite lipid monolayers of the membrane. In framework of our model, the tandem channel is represented as two dimers at the fixed distance 2*L*. The distance is determined by the length of the linker in the lateral dimer. The linker limits the maximal distance between the gramicidin A monomers and imposes some average separation between them. Thus, the linker length is denoted as the mean distance between the gramicidin monomers imposed by the linker rather than the contour length of its chain. This distance is determined by the contour length of the linker, its flexibility, conformational dynamics, its possible interactions with membrane and water, etc. However, the actual distance between gA molecules in the lateral dimer is not measured in the experiments [31,32,33]. Therefore, we considered the distance as a parameter and calculated the dependence of the elastic energy of the membrane on this parameter (Figure 4a,b). In this sense, the peptides considered in the model are not attached to the linker (as shown at the top of the right panel, Figure 4), as the linker is not explicitly accounted in our elastic model. When building the corresponding plots (Figure 4a,b), we only set the distance 2*L* and calculated the elastic energy, ignoring the driving forces behind the maintaining the particular distance. The pictures at the bottom of the right panel in Figure 4 are presented solely for illustrative reasons, in order to demonstrate how the distance between the dimers can be practically set by the linkers in the experimental systems [31,32,33].

The dissociation of one conducting dimer of the tandem channel transforms the configuration of two dimers to the configuration of the dimer and coaxial pair located at the same distance 2*L*. The elastic contribution to the energy barrier of dissociation, Δ*W*, is, thus, given as the difference between blue and green curves at a given distance 2*L* (Figure 4c).

Note that the dependences of the elastic energy *W*(2*L*) and Δ*W*(2*L*) do not account for the energy of H-bonds, which break upon dissociation of the dimer to the pair. This means that the actual “dimer-pair” curve should be shifted upwards by the energy of 4–6 H-bonds (~20–30 *k_B_T* [43]) with respect to the “dimer-dimer” curve. However, the energy of H-bonds is constant, i.e., it is independent from the distance between gramicidin A dimer and a coaxial pair. Thus, it makes sense to consider the relative change of the energy barrier of dissociation, i.e., the barrier at given distance 2*L* should be compared with the barrier, e.g., at infinite distance, keeping in mind that the actual energy barrier is positive.

Experimentally, the tandem channel has approximately doubled conductance as compared to the conductance of a single gA dimer [31,32,33]. The dissociation of one conducting dimer of the tandem channel is observed as a drop of the tandem channel conductance to approximately the value of the conductance of a single gA dimer [31,32,33]. From our results (Figure 4), it follows that the energy barrier of dissociation significantly depends on the distance between two conducting dimers, i.e., on the linker length in case of lateral dimers. For short linkers (left-most parts of plots in Figure 4a,b), the dissociation energy barrier is about 7 *k_B_T* higher than that of independent (non-interacting) dimers, both in membranes formed from DOPC and DPhPC. This should result in about *e*^7^ ≈ 1100 times longer lifetimes of the conducting state, provided that the linker does not disturb substantially the orientation of gA molecules in the lateral dimer. A successive increase of the length of the linker results in lower energy barriers of dissociation (Figure 4c), which should be observed experimentally as decreased lifetime of the conducting state. For the distances of about 4–5 nm, the energy barrier of dissociation is about 10 *k_B_T* lower as compared to the independent dimers. This means that for such linkers the tandem channel configuration characterized by approximately doubled conductance should be hardly observed, as the predicted lifetime of such state is extremely short.

The maximal distance of 20 nm between the molecules in the plots in Figure 4 was chosen quite arbitrarily, as at such (and larger) distances the membrane deformations, induced by two gramicidin A species (monomer/dimer/pair) in any combination, practically do not overlap, i.e., there are no membrane-mediated interactions. In this sense, the 20 nm distance plays a role in the effectively infinite separation, at which gAs can be considered as isolated. Note that for far separated dimers, the energy barrier of dissociation in membranes made from DOPC is about 2.5 *k_B_T* lower than that for DPhPC membranes (Figure 4c). This implies that the average lifetime of single gA channel should be substantially larger in DPhPC membranes as compared to DOPC membranes. This theoretical conclusion is in a qualitative accordance with available experimental data [19,21,32,33].

**Figure 5:**
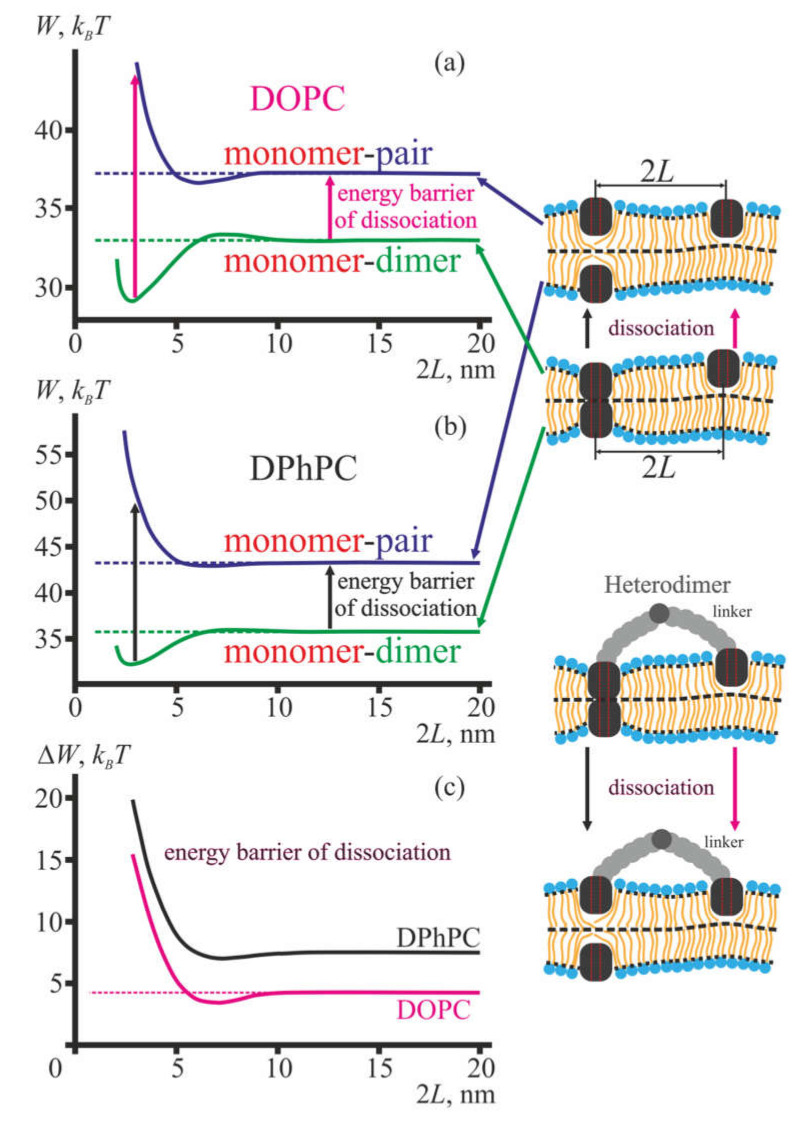
The energy of membrane elastic deformations induced by incorporated gramicidin A. Dependence of the membrane elastic energy on the distance between the gA monomer and gA dimer (green curve) and between the gA monomer and coaxial pair (blue curve) in membranes formed from DOPC (**a**) and DPhPC (**b**). (**c**) Dependence of the elastic contribution to the energy barrier of dissociation of the gA conducting state (dimer) on the distance between the conducting dimer and gA monomer; the dependences are obtained as a difference of blue and green curves in the plots (a) and (b) for membranes formed from DOPC (magenta curve) and DPhPC (black curve), respectively. The distances are measured between vertical axes of revolution of the molecules. Pairs of vertical red dashed lines on gA molecules schematically show the water pore inside them. The nearest possible distance between a dimer and a monomer, as well as between a pair and a monomer, equals to 2*r*_0_ = 2 nm.

Experiments can be carried out in asymmetric configuration: lateral dimers of gA are added to one side of the membrane, while gA monomers are added to the opposite side of the membrane [33]. Similar effects could be achieved if the surface density of gA is low enough and there is a population of gA molecules, which lack partners for the formation of the lateral dimer [31]. In this case, so-called heterodimers composed of the lateral dimer in one membrane leaflet and a single gA molecule in the opposing leaflet can be formed. We calculated the dependence of the elastic energy of membrane deformations induced by a transmembrane gA dimer (or coaxial pair) and gA monomer on the distance between axes of the monomer and the dimer/pair (Figure 5).

The obtained dependence of the elastic energy on the distance 2*L* imply that the conducting (transmembrane) dimer and a monomer are strongly attracted at short distances: the depth of the corresponding energy minimum is about 4 *k_B_T* (Figure 5a,b, green curves). If the approximation of pairwise interactions is valid, this should result in an elevated equilibrium concentration of monomers in the vicinity of the transmembrane dimer by about *e*^4^ ≈ 55 times. The interaction of the coaxial pair and gA monomer is strongly repulsive at short distances (Figure 5a,b blue curves). The elastic contribution to the energy barrier of the dissociation of the conducting state is determined as the difference between the energy of membrane deformations induced by the coaxial pair and transmembrane dimer. From the dependences illustrated in Figure 5a,b, we conclude that the elastic contribution to the energy barrier of channel dissociation increases with decreasing distance between the dimer and gA monomer (Figure 5c). This means that the lifetime of the conducting state should grow up sharply if there is a gA monomer in the vicinity of the transmembrane dimer. In particular, this effect should take place for the configuration of the heterodimer, where the distance between the conducting dimer or coaxial pair and the monomer is strictly set by the linker (Figure 5, right bottom cartoon). This observation is in accordance with the data of the works [31,32,33]. Note that for far separated dimers and monomers the energy barrier of dissociation in membranes made from DPhPC is about 2.5 *k_B_T* higher than that for DOPC membranes (Figure 5c), analogously to the case illustrated in Figure 4c. This implies that the average lifetime of a single gA channel should be substantially larger in DPhPC membranes as compared to DOPC membranes. This theoretical conclusion is in a qualitative accordance with available experimental data [19,21,32,33].

**Figure 6:**
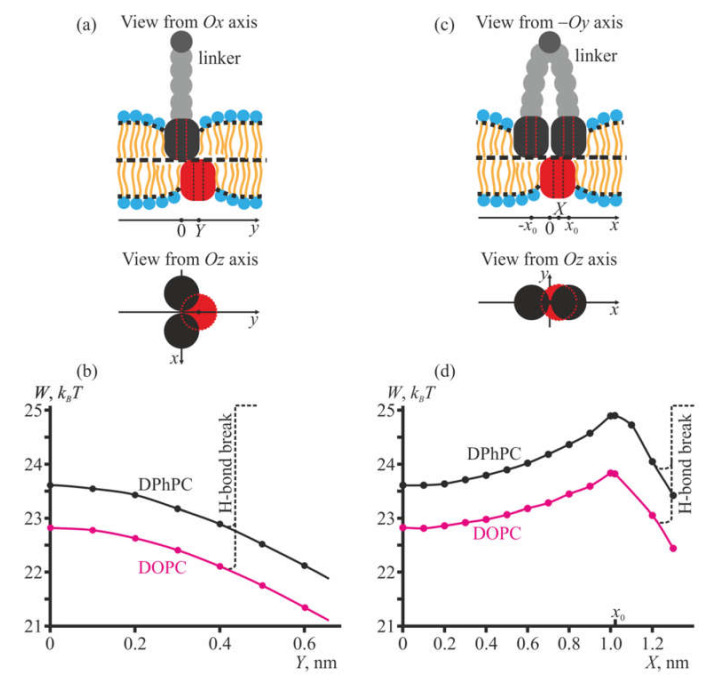
Dependence of the elastic energy of the membrane on the position of a single gA molecule in the heterodimer. gA molecules belonging to the lateral dimer are shown as black rectangles (side view) or black circles (top view); a single gA molecule in the heterodimer is shown as a red rectangle (side view) or a large red circle (top view). (**a**), (**b**) gA molecule is shifted in the direction of *Oy* axis; (**a**) side and top views of the configuration; (**b**) dependence of the membrane elastic energy on the coordinate *Y* of the center of the single gA molecule for membranes formed from DOPC (magenta curve) and DPhPC (black curve). (**c**), (**d**) gA molecule is shifted in the direction of *Ox* axis; (**c**) side and top views of the configuration; (**d**) dependence of the membrane elastic energy on the coordinate *X* of the center of the single gA molecule for membranes formed from DOPC (magenta curve) and DPhPC (black curve). The energy was calculated for the positions of a single gA molecule shown as small filled circles in (**b**) and (**d**). At some lateral shift of the single gA molecule, H-bonds formed with gA molecules of the lateral dimer have to break. The estimated positions corresponding to the breaking of one H-bond is shown as vertical dashed lines in panels (**b**) and (**d**).

In the works [32,33], it is observed that heterodimers (or configurations analogous to heterodimers) may manifest burst or flicker electrical activity. It seems probable that such an activity arises when a single gA molecule in the heterodimer can shuffle laterally between the gA molecules composing the lateral dimer. Upon such shuffling, if, e.g., the single gA shifts from the left gA of the lateral dimer to the right one, H-bonds between the single gA molecule and the left gA of the lateral dimer should break, but new H-bonds between the single gA molecule and the right gA of the lateral dimer should form. Therefore, the total number of H-bonds formed by the single gA molecule remains approximately constant, provided that the gA–gA distance in the lateral dimer, as well as the lateral shift of the single gA, are not large. We calculated the elastic energy of the membrane for a discrete number of positions of the single gA molecule in the heterodimer (Figure 6) for membranes formed from DOPC and DPhPC. The distance between the centers (vertical axes) of gramicidin A molecules in the lateral dimer was set at 2*x*_0_ = 2.1 nm. Note that the elastic energy of the membrane is not additive, and cannot be separated by its source: elastic deformations are characteristic of the lipid membrane. The embedded peptides impose boundary conditions on the membrane deformations, and the membrane adopts the shape determined simultaneously by the whole set of the boundary conditions imposed by all peptides. At each position, the single gA molecule of the heterodimer experiences deformations induced by both peptides of the lateral dimer, and their contributions cannot be formally separated; the deformations at each point are not the sum of the deformations generated by each peptide.

The conducting configuration is achieved when the axis of the single gA molecule approximately coincides with the axis of either gA of the lateral dimer. This takes place when the coordinate of the center of the gA molecule is close to either (*x*_0_, 0) or (–*x*_0_, 0) (Figure 6a,c). However, the elastic energy of the membrane in these configurations has local maxima (Figure 6d). From the point of view of the elastic energy minimization, the single gA molecule tends to escape from the heterodimer in the direction of the *Oy* axis (Figure 6b). However, a large lateral shift of the molecule should lead to breaking and, consequently, to the decrease of the total number of H-bonds formed by the molecule with gAs belonging to the lateral dimer. As H-bonds form between backbones of amino acid residues of two gAs located in opposing membrane leaflets, these bonds should be formed independently, one by one. For a rough estimation of the distance where one H-bond has to break, we assume that H-bond forming groups are homogeneously distributed along the perimeter of the first *β*−helix turn of the gA molecule, and there are four such groups in the turn (i.e., four H-bonds can be formed). Thus, one H-bond forming group occupies roughly 1/4 of the *β*-helix turn perimeter. We do not suppose that a specific orientation is necessary to form the H-bond between two gA molecules, but assume that solely bringing N-termini of two gAs in a close proximity is sufficient for H-bond formation. Analogously, separating the first turn of *β*-helices of two opposing gAs (either laterally or normally) by ~0.15 nm is considered to lead to an H-bond break. Assuming that one H-bond has to break if the distance between the 1/4 part of the perimeter of the single gA molecule and the perimeter of the gA belonging to the lateral dimer exceeds 0.15 nm, we come to the estimation that the threshold shift in the direction of the *Oy* axis is about *Y* = 0.4 nm, and in the direction of the *Ox* axis this is about *X* = 1.25 nm. Larger shifts along the axes should result in breaking of one H-bond, thus leading to abrupt growth of the energy of the system by about 5 *k_B_T* [43] (Figure 6b,d). From such reasoning, it follows that in the heterodimer the optimal position of the single gA molecule is given by coordinates *x* = 0, *y* = ±0.4 nm. To achieve the conducting configuration, the single gA molecule first has to stand in the position *x* = *y* = 0, surmounting the energy barrier of the height of about 0.5 *k_B_T* (Figure 6b). Furthermore, the molecule has to get the coaxial configuration with the gA molecule belonging to the lateral dimer, i.e., it should achieve the position *x* = ±*x*_0_ = ±1.05 nm, *y* = 0, in addition spending about 1 *k_B_T* (Figure 6d). Thus, the total energy necessary to transform from optimal configuration to the conducting one is about 1.5 *k_B_T*. This energy is relatively low, meaning that the single gA molecule should frequently achieve the conducting state as well as frequently escape from it. The total average duration of the burst or flicker activity is determined by the average lifetime of the heterodimer, which substantially exceeds that of a single transmembrane dimer of gA [32,33].

## 4. Discussion

In the present work, we analyzed how membrane-mediated interactions of gA monomers, coaxial pairs and transmembrane dimers influence the properties of channels formed by gA or its analogues. In all these configurations, elastic deformations of the membrane arise [16]. When the monomer, coaxial pair or transmembrane dimer are far separated, the induced deformations are independent and the elastic energy is additive. In the framework of the theory of elasticity of liquid crystals adapted for lipid membranes, we calculated the dependence of membrane elastic energy on the distance between two dimers, dimer and coaxial pair, dimer and monomer, coaxial pair and monomer of gA. In all cases, the elastic energy manifested non-monotonous oscillating behavior for separation distances below 10 nm, with the most pronounced effects for separations 2*L* < 5 nm (Figure 4a,b, Figure 5a,b). Such distances correspond to a gA surface density of an order of about 10^–2^ gA/lipid, while the usual surface density in experiments where electrical activity of only one to several channels is determined is about 10^–7^ gA/lipid [32]. The high surface density of gA molecules necessary for effective interactions was considered to be achieved by the use of a flexible linker binding two gA molecules in the same membrane leaflet, thus combining them into a lateral dimer. These linkers may differ by their chemical nature, length and flexibility [31,32,33]. The ion-conducting configurations involving lateral dimers are tandem channels (Figure 4) and heterodimers (Figure 5). In both configurations, elastic deformations of the membrane are induced; the energy of deformations contributes to the overall energy landscape of the gA ensemble. In particular, from the analysis of the elastic contribution to the energy barrier of dissociation of both heterodimer and tandem channel (Figure 4c, Figure 5c), it follows that the lifetime of these conducting states should exceed the lifetime of a single transmembrane dimer of gA by about 1000 times. This is in quantitative agreement with the data of the works [31,32,33]. Such durable states may be formed when the gAs in the lateral dimer are very close to each other, i.e., when the linker is rather short. In the work [32], the proximity of gA molecules in the lateral dimer is inferred based on an anomalous conductivity of transition states of formation and decay of the tandem channel. The observed conductivity is conformed to an increased dielectric constant of the immediate environment of the channel, which may take place if two transmembrane dimers of the tandem channel are close to each other. However, lateral dimers with too short linkers (15 vs. 23 amino acid residues) do not form tandem channels [32], although they are able to form single channels. We speculate that the short linkers disturb orientation of gA molecules in the lateral dimers in such a way that the first formed single channel prevents the formation of the channel by another pair of gAs, and thus, two lateral dimers located in opposing membrane monolayers form only a single channel rather than the tandem channel. Moreover, the folding of subunits in the lateral dimers with a short linker seems to be compromised: subunits of the 15-residue-linked tandem gA have an increased fraction of non-*β*^6.3^-helical structures [32]. Thus, the formation of durable tandem channels may take place in a relatively narrow range of linker lengths.

A similar approach has been used to investigate how elastic deformations of the membrane influence the gating properties of the mechanosensitive ion channel Piezo1 [44]. In this work, the energy of the system (protein, embedded into the membrane) is divided into the energy of the membrane deformations and the energy of the protein, which does not depend on the membrane shape. This allows investigating the influence of membrane deformations on the energy landscape of protein conformations.

Under high surface density conditions, lateral dimers of gA could principally form multiple channel networks. However, although the lateral dimers allow achieving a high local surface density of gA molecules, the average surface density of the lateral dimers is usually very low. In the works [31,32,33], as a rule, only one tandem channel is observed at a time. This means that the concentration of lateral dimers is roughly similar to the typical concentration of unmodified gA in single channel experiments. This concentration is estimated as 10^–6^–10^–7^ gA/lipid [32], meaning that the average lateral distance between two lateral dimers is about [(10^6^–10^7^) × 0.65 nm^2^]^1/2^ ≈ 800–2500 nm (0.65 nm^2^ is the area per lipid molecule). Under such conditions, the probability of formation of a multiple channel network seems very low. For increased concentration, the probability of multiple channel formation should rise. However, modeling of such a process is not quite straightforward, as the system of multiple lateral dimers possesses extremely large number of degrees of freedom, and finding its elastic energy minimum does not seem trivial.

To obtain quantitative results in the framework of our elastic model, we need to know the elastic and structural parameters of the membranes: the monolayer hydrophobic thickness, the values of the elastic moduli, the spontaneous curvature of the lipid monolayer, the membrane lateral tension and the area per lipid molecule. More accurate values of the parameters should provide more precise descriptions and predictions of the model. Moreover, the change of quantitative values of the membrane parameters may alter the qualitative behavior of membrane inclusions. For example, the mutual attraction of gA monomer and dimer (illustrated in Figure 5a,b) results from damped oscillations of membrane deformations, formally arising from the condition of local volumetric incompressibility, Equation (2). However, for larger splay modulus, *B*, or smaller tilt modulus, *K_t_*, the deformations become monotonous, i.e., they do not oscillate [45]. This should lead to qualitative switching of the dimer-monomer mode of interaction. Thus, a detailed experimental structure of a native lipid bilayer may improve the model. A striking example, when the detailed knowledge of a lipid bilayer structure provided a qualitatively improved model, is presented in the work [46]. In this work, a method of detergent-free extraction of the channel protein is utilized. It appears that the use of detergent in the course of protein extraction disrupts a patch of the lipid bilayer in the channel lumen, which remains intact under conditions of detergent-free extraction. The experimentally determined structure of the lipid bilayer patch allows proposing qualitatively an improved mechanism of the channel function.

In the works [32,33], it is observed that heterodimers manifest a burst or flicker electrical activity. Al-Momani and colleagues [33] explained such an activity, implying that the single gA rapidly exchanges partners for transmembrane dimerization between two gA molecules belonging to the lateral dimer. We analyzed the elastic energy landscape of lateral shuffling of the single gA molecule in the heterodimer. It appeared that the shuffling should not be associated with surmounting of high energy barriers, and, in principle, the partner exchange in conducting heterodimer may take place with high frequency. The total average duration of the burst or flicker activity is determined by the average lifetime of the heterodimer, which substantially (~1000 times) exceeds that of a single transmembrane dimer of gA. These conclusions are in agreement with the data on heterodimer conductance obtained in the works [31,32,33]. It is noteworthy that the presented consideration of the energy landscape of shuffling of a single gA molecule inside the heterodimer is rather qualitative and rough, as fine molecular details cannot be described in the framework of our continuum approach. Based on the calculation results illustrated in Figure 6, we can only conclude that the alteration of the membrane elastic energy upon the shuffling does not lead to large energy barriers. However, arising energy barriers may have a non-elastic nature. For example, if the distance between gA molecules in the lateral dimer is larger than about 0.5 nm, then the shuffling of the single gA molecule between gA molecules of the lateral dimer will most probably require the breaking of at least one H-bond between the single gA and either gA of the lateral dimer. The associated energy penalty should prohibit the shuffling, and thus exclude the observation of the burst or flicker electrical activity of the heterodimers; however, the landscape of the elastic energy of the membrane should change but slightly for 2*x*_0_ = 2.5 nm as compared to the case of 2*x*_0_ = 2.1 nm, illustrated in Figure 6. Heterodimers with an almost absent burst or flicker activity are observed in the work [31].

Previously, an elastic model that allows accounting for the elastic contribution to the energy landscape of gramicidin A dimerization/dissociation has been developed in the works [20,47]. In the framework of the model, it is assumed that elastic deformations of the membrane are induced by the gA transmembrane dimer only, while the coaxial pair and gA monomer do not deform the membrane. From the condition of hydrophobic matching, i.e., that the membrane thickness should match the length of the gA transmembrane dimer at its boundary, Hook’s law allows obtaining immediately that the elastic energy should be proportional to (*d*_0_–*l*)^2^, where *d*_0_ is the equilibrium thickness of the unperturbed bilayer core and *l* is the length of the gA transmembrane dimer [20]; *d*_0_ = 2*h*, *l* = 2*h_p_* in our notations. The proportionality coefficient is referred to as the spring constant; its numerical values are determined for membranes with particular lipid compositions [20,32]. The calculated energy penalty to dimerization of gA arising from deformations of the membrane is shown to strongly depend on the condition imposed on the contact angle of the membrane surface at the dimer boundary [47]. Two specific cases are usually distinguished: (i) free contact angle, corresponding to zero net torques at the dimer boundary; (ii) zero contact angle, i.e., the membrane surface is considered to be perpendicular to dimer axis of revolution. Using the latter boundary condition, Partenskii and colleagues generalized the linear spring model [20,47] for the case of multiple membrane inclusions in order to describe the increased lifetimes of gramicidin A tandem channels [48]. Formally, the deformation of lateral compression-stretching and the lateral tension of the membrane are taken into account in the work [48], although the authors referred to the contribution of the lateral tension as bending. It is shown that the energy of multiple inclusions can be expressed as the energy of coupled harmonic oscillators, and numerical values of the effective spring constants are obtained [48]. As bending deformation along with the volumetric incompressibility of the membrane are not taken into account, the obtained dependence of the spring constants on the distance between the inclusions is monotonous rather than damped oscillating (compare with Figure 4a,b,Figure 5a,b). Note that the non-monotonous profile of the interaction energy of two gA dimers calculated in the present work (Figure 4a,b, green curves) is in a good agreement with the analogous profile obtained by means of molecular dynamics in the work [15]. Under the condition of a zero contact angle, the close contact of two gA dimers is energetically favorable, as the elastic energy is lower than in the case of two far separated dimers. This formally explains the increased lifetimes of tandem channels [48]. However, such an explanation fails if the contact angle deviates from zero by even a small value, as the deviation immediately switches the interaction of two dimers to repulsion [49]. In the work [49], the condition of free contact angle is used, and it is not required that the angle should be constant along the dimer boundary, i.e., an anisotropic contact angle is allowed. Such boundary conditions qualitatively yield the attraction of two membrane-spanning cylindrical inclusions, and, generally, the model predicts aggregation (or clustering) of multiple inclusions [49]. However, in order to achieve quantitative agreement with the data on the lifetime of a single gA dimer, an assumption on hardening of the membrane in the dimer vicinity has to be done [50]. The hardening is shown to be mostly pronounced for the modulus of lateral compression-stretching, for which the calculated hardening coefficient is about 4.5. Although supported by elegant mathematical treating, such mostly artificial assumptions are not needed if the elastic energy landscape is described in the framework of more detailed theories of elasticity [34,35]: corresponding models will allow achieving quantitative agreement on the dependences of the channel lifetime and formation probability on the elastic parameters of the membrane, even if the parameters are considered as laterally uniform [16]. Moreover, the assumption that only transmembrane dimers of gA deform the membrane excludes any possibility of membrane-mediated interactions of gA dimer and monomer, dimer and coaxial pair, coaxial pair and monomer. However, accounting for such interactions is critical to explain the increased lifetime of heterodimeric channels (Figure 5), observed experimentally [31,32,33].

To conclude, in the present work based on the theory of elasticity of lipid membranes, we developed a quantitative model which allows explaining experimentally observed phenomena under conditions of the high surface density of gramicidin A or its analogues. The model could be further used for the analysis and prediction of the gA electric response in various experimental conditions: in the wide range of gA surface density, in the presence of membrane-deforming inclusions (e.g., amphipathic peptides), in membranes of various lipid compositions, etc. This potentially widens the possible applications and ways of regulation of gA as a molecular sensor of membrane elasticity [22].
